# FDA Approved Biologics: Can Etanercept and Ustekinumab be Considered a First-Line Systemic Therapy for Pediatric/Adolescents in Moderate to Severe Psoriasis? A Systematic Review

**DOI:** 10.7759/cureus.9812

**Published:** 2020-08-17

**Authors:** Nida Aslam, Hajra Saleem, Salikh Murtazaliev, Sohail J Quazi, Safeera Khan

**Affiliations:** 1 Dermatology, California Institute of Behavioral Neurosciences & Psychology, Fairfield, USA; 2 Family Medicine, California Institute of Behavioral Neurosciences & Psychology, Fairfield, USA; 3 Internal Medicine, California Institute of Behavioral Neurosciences & Psychology, Fairfield, USA; 4 Plastic Surgery, California Institute of Behavioral Neurosciences & Psychology, Fairfield, USA; 5 Plastic and Reconstructive Surgery, Hamad Medical Corporation, Doha, QAT

**Keywords:** psoriasis, pediatric, etanercept, ustekinumab

## Abstract

Psoriasis is a chronic immune-mediated skin disorder. Due to lack of clarity in its pathogenesis, a cure with existing treatment is a big challenge. Biologics, a revolutionary treatment, are potent immunomodulators that explicitly target the culprit cells of the immune system to achieve the maximum level of Psoriasis Area and Severity Index (PASI) score (75 to 90) and clear or almost clear skin in moderate to severe psoriasis. They have been a successful therapy in adult severe psoriasis for a decade.

In recent years, biologics have unprecedently sought the attention of the pediatric psoriatic population by proving an efficacious and safe option. The aim of the study is to provide a systematic review of efficacy, safety, and impact on the quality of life of Food and Drug Administration (FDA)-approved biologics, namely etanercept and ustekinumab, and their use as a “first-line systemic therapy” in the moderate to severe pediatric and adolescent psoriatic population.

We explored PubMed, Cochrane Library, Google Scholar, American Academy of Dermatology website, ClinicalTrials.gov, the FDA site, and the National Psoriasis Foundation USA site as major database searches. Psoriasis, pediatric, etanercept, and ustekinumab were keywords used to find the relevant literature. Clinical trials and observational studies were retrieved and analyzed to assess the efficacy and safety of FDA-approved biologics as first-line systemic therapy in pediatric psoriasis.

The relevant evidence-based studies and the Joint American Academy of Dermatology-National Psoriasis Foundation (AAD-NPF) guideline have shown that etanercept and ustekinumab biologics are significantly effective and safe systemic therapies in dealing with moderate to severe psoriasis in pediatric and adolescent patients and have unprecedently improved their quality of life. Thus, they can be confidently considered as first-line systemic therapy in moderate to severe pediatric and adolescent psoriatic patients by applying the specific criteria and proper monitoring. However, health practitioners and dermatologists must educate pediatric patients and their caretakers about their adverse effects, success/failure chance, careful monitoring, and follow-up plan to achieve the desired result.

## Introduction and background

Psoriasis is a complex, chronic, autoimmune, genetic disorder with characteristics of well-defined scaly plaque with mild itching [1]. The etiology of psoriasis is yet to be established, but histological examination of its lesions indicates the association of innate and adaptive immunity. The dermis and epidermis are the main culprits in an inﬂammatory infiltrate of T-lymphocytes, mast cells, neutrophils, and macrophages, which leads to hyperkeratosis, epidermal acanthosis, parakeratosis, and amplification of the rete ridges [2].

Epidemiologically, two to five percent of the total population is hit by psoriasis worldwide, and one-third of the cases start from childhood [3]. The incidence of pediatric psoriasis is 40.8 per 100,000, which is remarkably less than adult psoriasis (78.9 per 100,000) [4].

A long-lasting disorder like psoriasis majorly affects the patient's self-esteem, physical appearance, and self-image. Thus, it causes detrimental effects (depression, anxiety, and suicidal thoughts) on the psychological and physical well-being of human beings [5].

The most common type that affects the majority of the adult and pediatric population is plaque psoriasis/psoriasis vulgaris [4]. Psoriasis can also involve other comorbidities, including cardiac problems, diabetes, arthritis, obesity, hypertension, arthritis, inflammatory bowel disease, and lipid dysregulation [3]. Psoriatic arthritis coexists most frequently in psoriatic patients. Noticeably, 80% of pediatric patients acquire psoriatic arthritis before cutaneous manifestation [6].

In the majority of diagnoses, psoriasis is confirmed by physical examination, though biopsy can be required in some ill-defined clinical presentation [7]. However, children with psoriatic lesions do not show severity in symptoms in contrast to the older population. They are also approached differently to cope with psychological and physical morbidity [8].

The management of moderate to severe pediatric psoriasis has always been complex due to the absence of a complete cure. Therefore, the purpose of treatment is the remission of the disorder by alleviating the symptoms with clear/almost clear skin. A breakthrough occurred in 2019, where a guideline for pediatric psoriasis was formulated, which showed the efficacy and safety of few biologics, mainly etanercept and ustekinumab.

Systemic therapies: biological versus non-biological

The pediatric population with moderate to severe psoriasis mainly requires systemic therapies, for example, cyclosporin, methotrexate, cyclosporine, acitretin, phototherapy, and biologics [9].

The use of traditional/non-biological systemic therapies including phototherapy and immunosuppressants such as cyclosporin, methotrexate, and acitretin has been common in moderate to severe pediatric psoriasis for decades [10]. These should be cautiously used with proper liver function, renal function, and blood pressure monitoring. The most common immunosuppressant which is prescribed by practitioners is methotrexate for moderate to severe cases in children [11]. The guidelines for conventional/non-biological systemic treatment are mainly derived from adult psoriasis guidelines, limited pediatric studies, and expert consensus [12]. These non-biological systemic therapies lack significant evidence-based literature, and more work needs to be done as it's an hour of need to provide the safest option with better efficacy in pediatric psoriasis.

Biologics are immunomodulators that regulate the inflammatory pathway and are widely considered for moderate to severe psoriasis due to their specification on inflammatory cells. Moreover, they do not show the toxicity/serious adverse effects like other conventional systemic therapies and have a more appropriate fixed dosing plan with less frequent follow-up schedules.

In recent years, only two biologics have been approved by the Food and Drug Administration (FDA) for pediatric moderate to severe plaque psoriasis, namely etanercept and ustekinumab. Etanercept is a human recombinant tumor necrosis factor receptor fusion protein that acts as a tumor necrosis factor (TNF) inhibitor. Ustekinumab is a human monoclonal antibody that antagonizes the p40 subunit of interleukin-12 (IL-12) and interleukin-23 (IL-23).

Biological systemic treatment aims to either eradicate/suppress the disease or manage its stability for an extended period by fixing the dose to the lowest level with the least toxicity. Once the severity of the disease is controlled, it is suggested to stop the systemic treatment. Above all, the rule of thumb is always to select a drug with fewer side effects if long-term therapy is required [13].

Critical approach towards biologics

Treating the pediatric moderate to severe psoriatic population with biologics is a big challenge but as per the Joint American Academy of Dermatology-National Psoriasis Foundation (AAD-NPF) guideline: severity of illness, failure of other traditional treatments (topical and other systemic medication, phototherapy), compromised psychosocial quality of life, comorbidities mainly arthritis and types of psoriasis are the key indicators for selection of biologic treatment [13].

## Review

Method

We conducted this systematic review in the light of Preferred Reporting Items for Systematic Review (PRISMA) guidelines. For data collection, we explored various search engines such as PubMed, Cochrane Library, Scopus, Google Scholar, MEDLINE, PubMed Central (PMC), the official American Academy of Dermatology website, Nice guidelines, the official website of clinical trials (ClinicalTrials.gov), the FDA site and non-profit organization, and the National Psoriasis Foundation USA site. We conducted our search by applying Medical Subject Heading (MeSH) terms and keywords, which were "psoriasis," "pediatric," "etanercept," and "ustekinumab." Search strategy was ("Psoriasis"[MeSH]) AND ("pediatrics/etiology"[MeSH] OR "pediatrics/immunology"[MeSH]) AND ("etanercept/administration and dosage"[MeSH] OR "etanercept/adverse effects"[MeSH] OR "etanercept/therapeutic use"[MeSH]) OR ("ustekinumab/administration and dosage"[MeSH] OR "ustekinumab/adverse effects"[MeSH]). We collated papers from the past 10 years without any gender bias. Studies were mainly based on clinical trials and retrospective cohort studies and were assessed for quality and were peer-reviewed. Relevant and long-term based studies were less in this population due to the age factor. Meanwhile, in a few studies, biologics were compared against other systematic therapies, mainly methotrexate.

**Figure 1:**
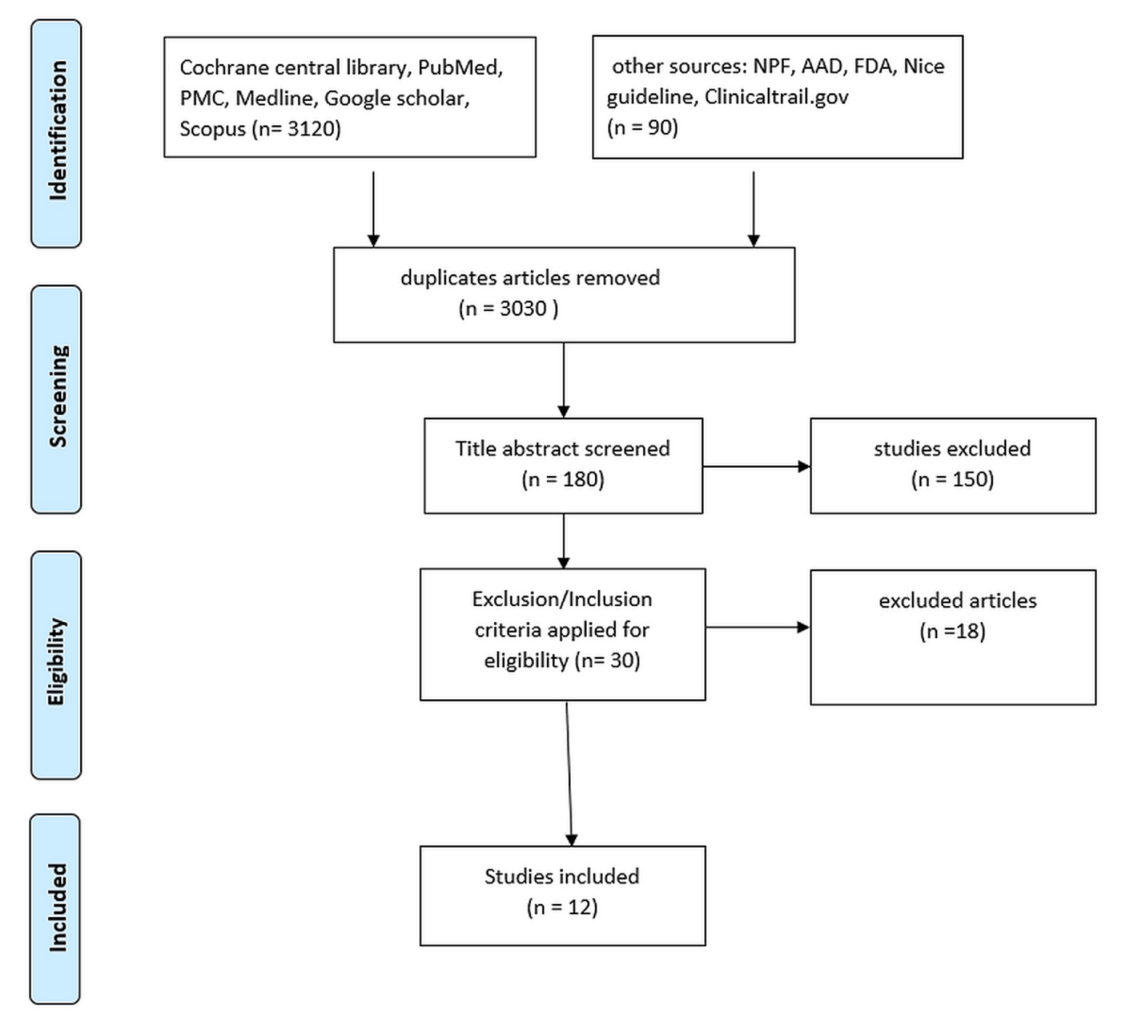
PRISMA flow diagram PMC: PubMed Central, AAD: American Academy of Dermatology, NPF: National Psoriasis Foundation

The exclusion and inclusion criteria were applied. Out of 3210 studies, we retrieved only 12 studies that focused on the pediatric population and related to humans only and were in the English language. Eligible patients were 17 years old or under with moderate to severe chronic/unstable plaque psoriasis with a duration of ≥six months having minimum Psoriasis Area and Severity Index (PASI) score of 10 (index range = 0-72, while maximum range depicts the severity of disease), and Physician Global Assessment (PGA) criteria should be at least three (zero = clear, five = worsening of disease). The Body Surface Area (BSA) of psoriasis involvement was equal to or more than 10%. We excluded all other types of psoriasis except plaque psoriasis. The PRISMA diagram is shown in Figure 1 [14].

Results

Out of the 3210 studies, 12 were selected after a quality check. Studies were mainly comprised of clinical trials and observational studies, while a few case series were also included. Etanercept had the highest level of evidence support, then ustekinumab, as etanercept is an older agent that got approval in 2015 for the pediatric psoriatic population. While ustekinumab has been recently approved by the United States Food and Drug Administration for the pediatric population and needs more attention to get a large pool of data, each of these studies was analyzed in terms of inclusion and exclusion criteria.

The primary aim of all studies was to assess the safety and efficacy of FDA approved biologics, namely etanercept and ustekinumab, on plaque psoriasis/psoriasis vulgaris in the pediatric/adolescent population. The secondary aim was to prove it as a "first-line systemic agent" and show its dominance over conventional systemic therapies in moderate to severe pediatric psoriasis.

Data of the selected studies are gathered and documented in Table 1.

**Table 1:** Data from the studies with the conclusion PASI: Psoriasis Area and Severity Index

AUTHOR AND YEAR OF STUDIES.	TYPES OF STUDY	NO OF PATIENTS	STUDY PURPOSE	CONCLUSION/RESULT
Paller et al., 2010 [15]	clinical trial	182	To determine the efficacy and safety of etanercept in pediatric psoriasis.	Etanercept was safe and effective in the pediatric population.
Paller et al., 2015 [16]	clinical trial	182	To find the safety and efficacy of etanercept in pediatric psoriasis.	Etanercept was well tolerated and effective in pediatric psoriatic patients.
Siegfried EC, 2010 [17]	clinical trial	138	To evaluate the efficacy and safety of etanercept by injecting intermittently in the psoriatic pediatric population.	Etanercept therapy appeared safe by injecting intermittently in pediatric. 80% patients achieved PASI 75 without showing any serious side effects.
Langley RG, 2010 [18]	clinical trial	211	To assess the health-related quality of life Impact of etanercept versus placebo in psoriatic pediatric patients.	Health-related quality of life (hrqol) was significantly improved in the etanercept's group as compared to the placebo.
Di Lernia V, et al., 2017 [19]	Observational study	23	To evaluate efficacy, tolerance, and discontinuation reason of etanercept in the pediatric population.	Etanercept monotherapy was efficacious and well-tolerated in real-life cohort pediatric patients.
Landells I, 2015[20]	clinical trial	110	To determine efficacy and standard safe dose of ustekinumab without any adverse effects in adolescents (12-17 years) psoriatic population.	The standard dose (0.75 mg/kg) of ustekinumab with a bodyweight ≤60 was significantly effective in adolescent psoriatic patients without any hazardous adverse effects.
Bronckers IM, 2017 [21]	An observational study (cohort)	390	To evaluate the therapeutic use and relative risk of systematic agents including biological and non-biological therapies in pediatric psoriatic patients.	Biologic showed less adverse effects as compared to the conventional systemic agent (methotrexate). Out of 106, only four patients on biologics left treatment, while 44 pts from >370 discontinued conventional therapy due to ≥ one adverse effect.
Klufas DM, 2016 [22]	case series	51	To assess the better systemic drug therapy for moderate to severe psoriasis in pediatric patients.	It demonstrated that biological therapy was safe, well-tolerated as compared to traditional systemic therapies in moderate to severe psoriatic pediatric patients. It also showed the safety of combination therapy (biologics + methotrexate) and suggested that it can be used for better response in relapsing psoriasis and psoriatic arthritis.
Bronckers IM, 2020 [23]	Observational study	234	To find the psoriasis severity reduction in real-world practice and drug survival of biologics versus methotrexate in pediatric psoriasis.	Biologics were highly effective, safe, and had better drug survival in the long-term period as compared to methotrexate in pediatric psoriasis.
Phan C, 2019 [24]	Observational study	134	To assess the difference in the selection/outcome of biologic in real-life practice versus clinical trials.	This study suggests that the selection of biological therapy in real-life practice differs or hard to match from clinical trial’s criteria of biologics in the pediatric psoriatic population due to the strict eligibility parameters.

Discussion

**Figure 2:**
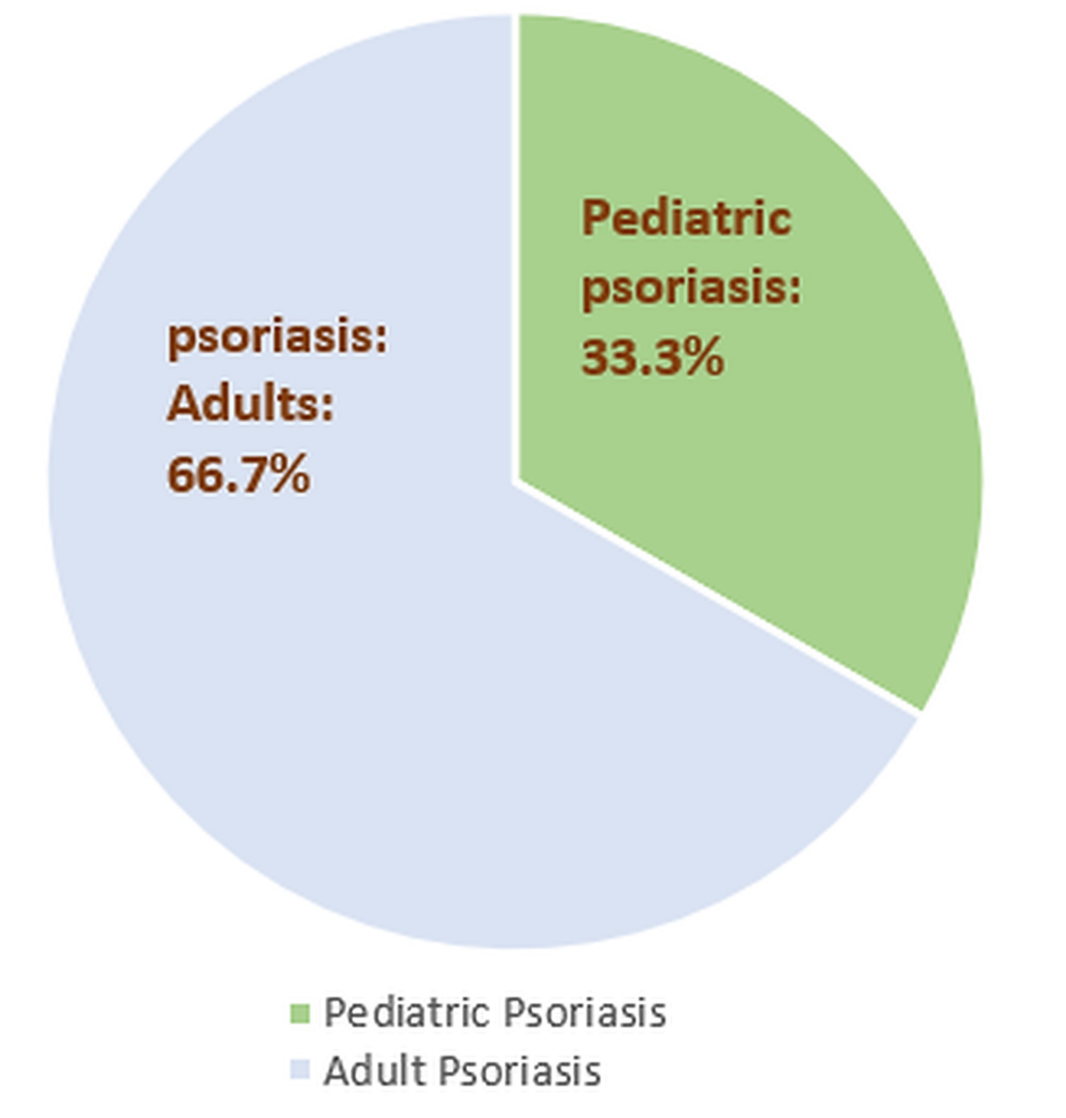
Age-wise distribution of psoriasis

Psoriasis is a chronic, inflammatory, immune regulated skin disorder that mostly occurs in adulthood (66.7%) and is less commonly found in childhood (33.3%) (Figure 2). However, one-third of psoriatic patients experience mild symptoms during childhood without knowing or diagnosing it. Most of the pediatric population acquire mild to moderate psoriatic symptoms, which can be easily tackled with a topical regimen and phototherapy sessions. The biggest challenge is for those who suffer from moderate to severe psoriasis where topical treatment and phototherapy often become toothless. Their ray of hope is wholly confined to systemic therapies, either non-biological or biological [25]. Biologics have shown dominance over non-biological therapies.

**Figure 3:**
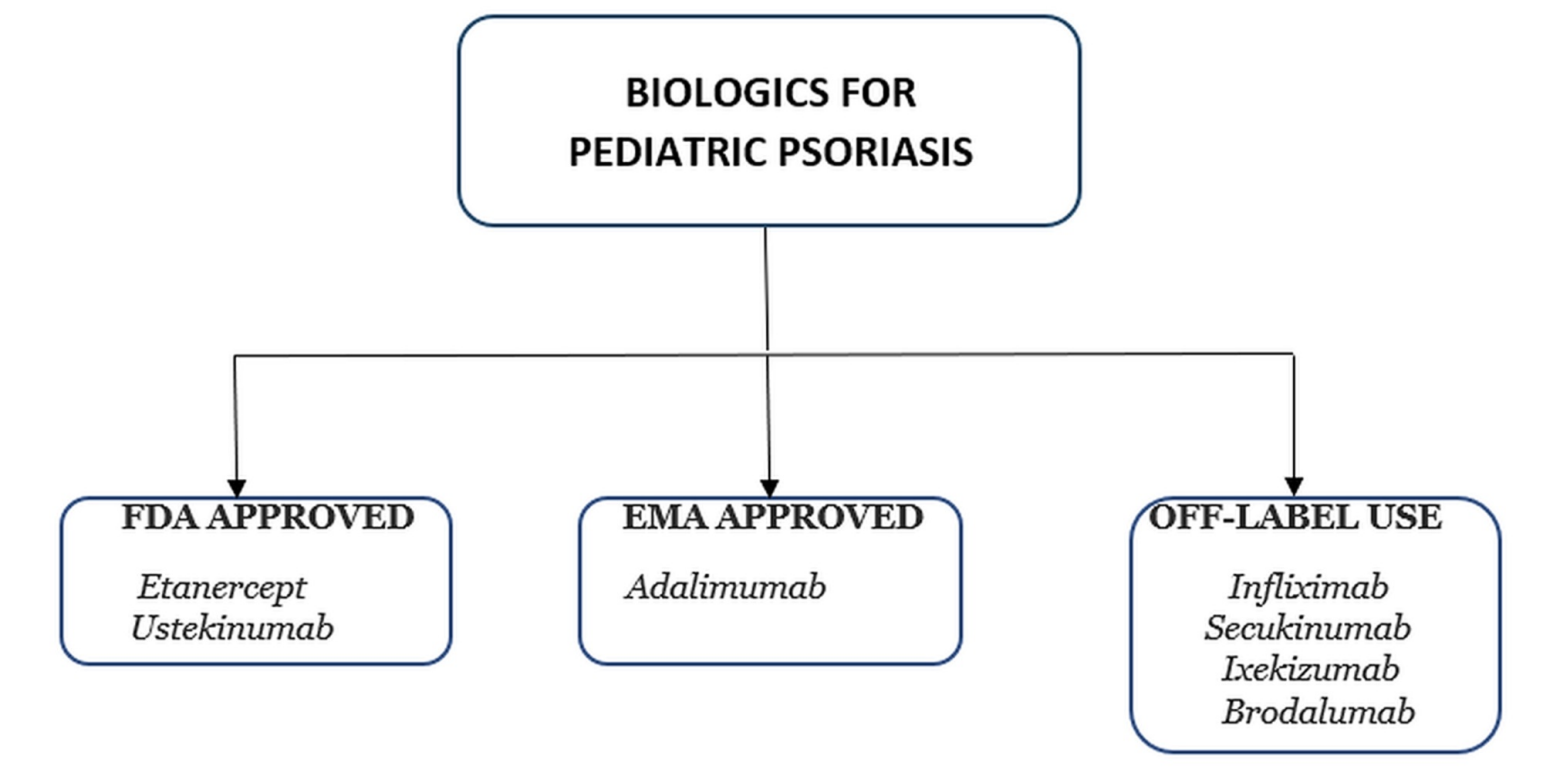
Biologics for the pediatric population of psoriasis FDA: Food and Drug Administration; EMA: European Medical Authority

By mid-2020, more than 14 biologics (tumor necrosis factor-alpha blocker, interleukin-12/23 and interleukin-17 inhibitors) have been approved by the FDA for adult psoriasis. On the contrary, only etanercept and ustekinumab have approval for the pediatric psoriatic population, although many other drugs are being used off-label. Figure 3 shows the total number of biologics for pediatric psoriasis.

**Figure 4:**
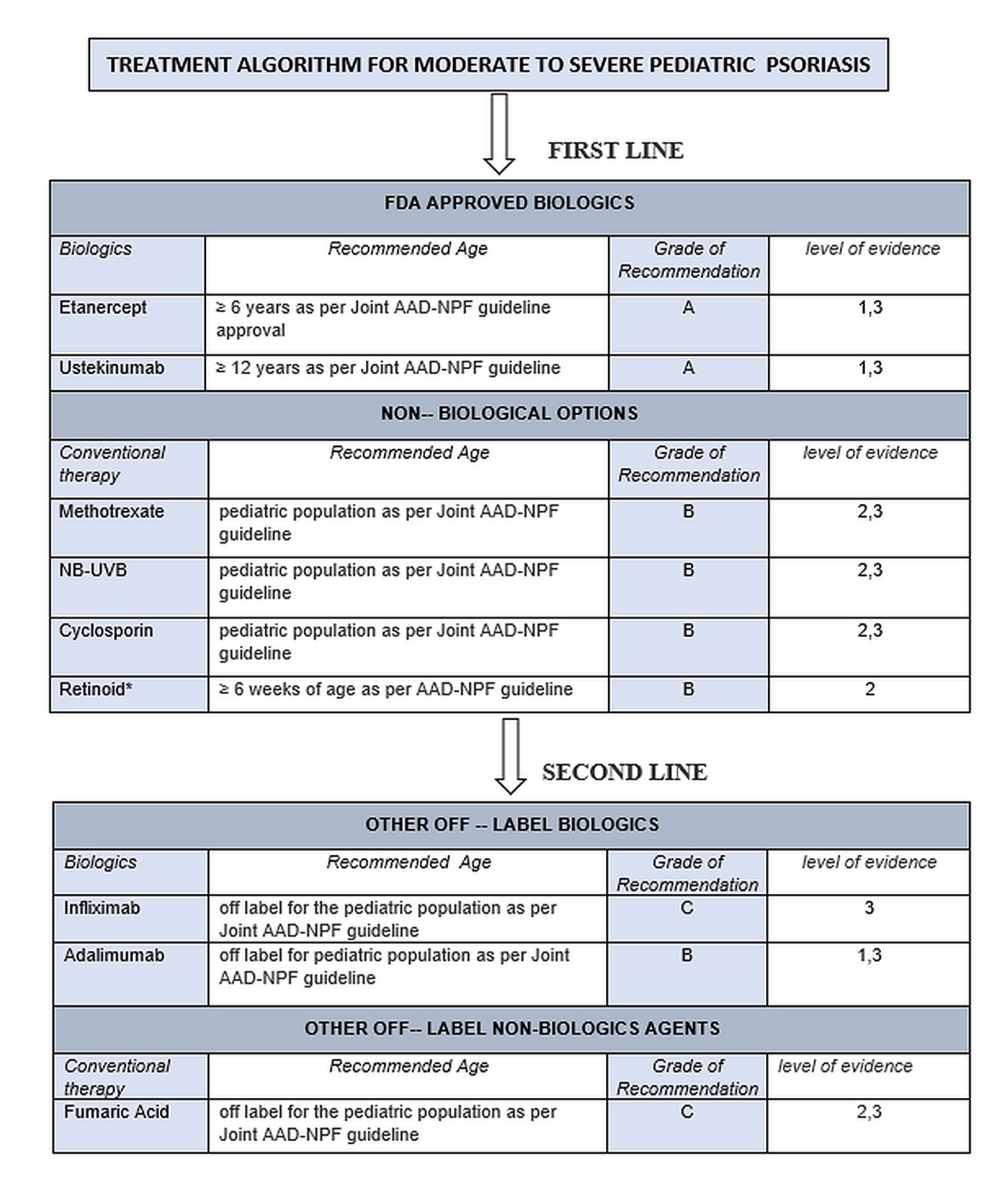
Treatment algorithm for the moderate to severe pediatric psoriasis according to Joint AAD-NPF guideline 2020 *all kind/types AAD-NPF: American Academy of Dermatology-National Psoriasis Foundation

Although methotrexate, cyclosporin, and phototherapy are efficacious for alleviating the severity of pediatric psoriasis, the long-term safety concerns and inconvenience due to lack of evidence-based studies on systemic therapies have led pediatric patients to consider biologics as first-line therapy [26]. The majority of conventional non-biological interventions (methotrexate, cyclosporin, acitretin) are being used off-label in moderate to severe pediatric psoriasis. Recently, with better knowledge of psoriasis etiology and having a large pool of clinical studies on the pediatric population, many biologicals interventions have been formulated (phosphodiesterase-4 inhibitor, biologics, and Janus kinase inhibitor) and have been proved as a great hallmark to achieve PASI 75 to PASI 90 and PASI 100 [25]. The treatment algorithm, as per the Joint AAD-NPF guideline for moderate to severe pediatric psoriasis, is shown in Figure 4.

Etanercept is a tumor necrosis factor-alpha blocker that has shown significant therapeutic potency in psoriasis. It has been approved for both adult and pediatric psoriasis for many years. The recommended age of its usage is six years or over. Several case series reported its efficacy even in 22-month-old children by administrating as a monotherapy/adjuvant therapy with other conventional regimes in moderate to severe plaque psoriasis, palmoplantar psoriasis, erythrodermic psoriasis, pustular psoriasis, erythrodermic psoriasis, and palmoplantar psoriasis [7]. The detailed pharmacologic summary of etanercept is given in Table 2.

**Table 2:** Summary of FDA approved biologics in pediatric psoriasis Adapted from Fortina et al. [12] TB: tuberculosis; HCV: Hepatitis C virus; HBV: Hepatitis B virus

DESCRIPTION	ETANERCEPT	USTEKINUMUMAB
FDA approval for Pediatric moderate to severe Psoriasis	yes	yes
Age	≥6 years	≥12 years
Working Mechanism	human fusion protein which combines with a soluble fusion protein of TNF-α	A human monoclonal antibody which binds to p40 Subunit of IL 12-23
Route of administration	subcutaneous	subcutaneous
Recommended baseline monitoring parameters	TB, HIV, HCV, HBV screening incl: chest radiographs	TB, HIV, HCV, HBV screening incl: chest radiographs
Recommended dose	0.8 mg/kg weekly . if ≥ 63 kg, 50 mg.	0.75 mg/kg/dose (<60 kg). 45 mg (60 to ≤ 1oo kg). 90 mg (>100 kg) by week 0, 4, and every 12 weeks.
Absolute contraindication	Active infections including active TB, hepatitis	Active infections including active TB, hepatitis
Adverse effects	Primary AE (injection site reaction), Infections	Primary AE (injection site reaction), Infections

Ustekinumab is a human monoclonal antibody that combines with the p40 subunit of interleukin-12/13 (IL- 12/23), which causes cessation of its receptor's function. It is recommended for adolescent and adult psoriatic patients [27]. In large, it has shown significant clinical response though partial response can also occur due to different triggered immune-mediated pathways other than interleukin 12 and 23 immune-mediated pathways [28]. Noticeably, in comparison to other biological agents, ustekinumab requires fewer injections, resulting in easy follow-up for patients [2]. Hence, the therapeutic option should be considered specifically patient-by-patient while taking into account the impact of the disease on the patient and their family [29]. The comprehensive pharmacological summary of ustekinumab is presented in Table 2.

A systematic review was conducted to evaluate the biologics as first-line systemic therapy for moderate to severe pediatric psoriasis by collating the data and experts' opinion in the presence of other conventional non-biological treatments, mainly methotrexate. This will assist the dermatologists in treatment selection with a better understanding in the light of more substantial evidence.

Under the clinical trial study in 2010, Paller et al. assessed the long-term safety and efficacy of etanercept by enrolling 182 patients, where 140 (76.9%) patients completed the therapy until 96 weeks and achieved Psoriasis Area and Severity Index 50 (89%), 75 (61%), and 90 (30%). The Physician Global Assessment was observed by 96 weeks, where 47% of pediatric patients got clear/almost clear skin. As far as safety concerns, five patients had mild adverse effects; the most frequent was respiratory tract infections (24.9%), and headache, sinusitis, and pharyngitis were also listed. Etanercept was generally effective and safe with no observed lethal infection, death, or malignancies [15].

Paller et al. (2015) further extended their clinical trial of 2010 to confirm the long-term efficacy and safety of etanercept in moderate to severe pediatric patients. Similar to the previous study, 182 patients were enrolled, where only 69 patients maintained by 264 weeks and secured PASI 75 (60-70%) and 90 (30-40%). The skin was clear/almost clear by 40-50% by following the Physician's Global Assessment criteria. As per safety status, 89% of pediatric patients reported mild adverse effects, most commonly upper respiratory tract (37.6%). Seven patients reported serious side effects, predominantly cellulitis. By and large, etanercept was considered as an effective and safe option in moderate to severe pediatric psoriasis [16].

Another most important clinical trial in 2010 by Seigfried et al. indicated the efficacy and safety of etanercept in the intermittent phase in pediatric psoriatic patients. One hundred thirty-eight patients were recruited and randomized into two groups (etanercept versus placebo) for 12 weeks. Seventy percent of patients on etanercept achieved PASI 75 compared to placebo (45%). While those who did not achieve PASI 75 in placebo were re-randomized and got open-label etanercept retreatment. The majority of them regained the PASI 75 after four to eight weeks. Furthermore, after completion of 48 weeks study, 80% of patients on etanercept maintained PASI 75 without showing any serious side effects. This complex treatment pattern supports etanercept's efficacy and safety in pediatric psoriatic patients in real-world settings [17].

In 2010, Langley et al. conducted a clinical trial in support of etanercept use in pediatric psoriasis patients. The center of the study was a quality of life impact being treated with etanercept therapy versus placebo. It mainly evaluated the Health-related Quality of Life (HRQoL) including emotional and psychosocial well-being and therapy effect of psoriasis in pediatric patients by providing the multi-colored cartoon-based questionnaire, Children's Dermatology Life Quality Index (CDLQI) and Pediatric Quality Of Life Inventory (pedsQL). Those who received etanercept therapy showed improved HRQoL with higher mean percentage improvement in CDLQI score (etanercept 52% versus placebo 17%) [18].

In support of another biological therapy, namely ustekinumab in moderate to severe psoriatic adolescent patients, Landell et al. (2015) conducted a double-blinded, placebo-controlled trial. It proved the efficacy and safety of ustekinumab by enrolling 118 adolescent psoriatic patients. It is seen that adolescents showed significant improvement with both half standard-dose (HSD) and standard-dose (SD) by observing the PASI 90 (HSD, 54.1%; SD, 61.1%, placebo, 5.4%) and PASI 75 (HSD, 78.4%; SD, 80.6%; placebo, 10.8%). The disease severity was markedly reduced with improved quality of life by 60 weeks. Eighty percent of patients turned up with at least one adverse effect, which was already calculated. Overall, it is considered one of the best therapies for moderate to severe psoriasis in adolescents with better efficacy and safety [20].

Bronckers et al. (2020) in a six-month retrospective cohort study demonstrated a comparison between biologics and methotrexate therapies in moderate to severe pediatric psoriasis population to evaluate the most potent therapeutic option. Under this study, 163 patients were treated with methotrexate, while 47 patients received biological therapy. Both groups showed significant improvement, but biologics were superior to methotrexate in terms of efficacy and safety. Most of the methotrexate patients left the study due to drug-related adverse effects (nausea, infection, and liver function dysregulation). At the same time, very fewer patients on biologics discontinued their treatment due to mild adverse effects [23]. 

To compare the safety of systemic therapies (biologics versus other non-biological systemic therapies, mainly methotrexate) in moderate to severe pediatric psoriasis, a retrospective study was conducted (Bronckers et al., 2017). In 390 patients, methotrexate was given to 270 children, biologics to 106 patients, and cyclosporine, fumaric acid, and acitretin were used in the rest of patients. Forty-eight percent of patients on methotrexate showed ≥one adverse effect, including gastric issues and laboratory test abnormalities, while 18% of children got injection site reactions during biologic administration. Hence it proves that medication-related adverse effects were less observed in biologics as compare to other systemic therapies [21].

With a different approach to biological therapies in pediatric psoriasis patients, Phan et al. (2019), under a cohort study, evaluated the eligibility criteria of biologics in real-life practice on children, which has been applied in phase three clinical trials of etanercept, adalimumab, and ustekinumab. A total of 134 patients were enrolled in this study, 73 of which (54%) became ineligible after following the criteria (PASI score ≥10, PGA > 3, other types of psoriasis, the coexistence of other therapies, and washout period) of clinical trials. This highlights that the eligibility criteria of childhood psoriasis in clinical trials are hard to match in real-life practice, as the main differential factors including PASI score (≥10 and PGA > 3), types of psoriasis (palmoplantar and guttate), and concomitant therapies are not reflected all together in real-life practice [24].

Based on the evidence-based literature, the FDA-approved biologics (etanercept and ustekinumab) were significantly effective and safe in moderate to severe pediatric psoriasis in comparison to other conventional systemic treatments and can be considered as first-line systemic therapy by following the laboratory/clinical monitoring and criteria of biologics.

Limitation

There is a paucity of high-quality studies on FDA approved biologics in the psoriatic pediatric population. Very few studies had extended trail/duration. These observations were pronounced in the case of ustekinumab. Many studies were compromised on the blinding process, indicative of a bias.

## Conclusions

From the above literature, it is concluded that etanercept and ustekinumab showed great efficacy, improved quality of life, and safety in moderate to severe pediatric and adolescent psoriatic patients. All studies reported that PASI 75 to 90 was achieved by a large pool of pediatric patients with clear or almost clear skin, even when compared with other traditional therapies, although more research needs to be done on the psoriatic pediatric population. Meanwhile, adverse effects were also noticed less in biologics than other conventional/non-biological systemic treatments (methotrexate, cyclosporin, and acitretin). The selection and monitoring of a particular biologic should be followed as per the current Joint AAD-NPF guideline to avoid unnecessary adverse effects/risks. A systematic review of the literature suggests that these biologics can be considered as a first-line systemic therapy option in the moderate to severe pediatric psoriatic population. Importantly, the advantages and side effects of biologics should be discussed with the respective patient/patient's caretaker. The patient's willingness and priority of treatment option should be taken into account.
